# Comparisons of Curative Effects of Chlorophyll from* Sauropus androgynus* (L) Merr Leaf Extract and Cu-Chlorophyllin on Sodium Nitrate-Induced Oxidative Stress in Rats

**DOI:** 10.1155/2016/8515089

**Published:** 2016-12-08

**Authors:** Suparmi Suparmi, Minidian Fasitasari, Martanto Martosupono, Jubhar Christian Mangimbulude

**Affiliations:** ^1^Department of Biology, Faculty of Medicine, Universitas Islam Sultan Agung, Semarang 50112, Indonesia; ^2^Department of Clinical Nutrition, Faculty of Medicine, Universitas Islam Sultan Agung, Semarang 50112, Indonesia; ^3^Sultan Agung Islamic Hospital, Semarang 50112, Indonesia; ^4^Master Program of Biology, Satya Wacana Christian University, Jl. Diponegoro 52-60, Salatiga 50711, Indonesia

## Abstract

Sodium nitrate (NaNO_2_) widely used as food additive for coloring and preserving meat has been reported to induce oxidative stress and cause histopathologic changes, nitrosative tissue damage, and lipid peroxidation in liver and kidney. Therefore, the present study compared the curative effect of chlorophyll from* Sauropus androgynus* (L) Merr and Cu-chlorophyllin as antioxidant in NaNO_2_-induced female Wistar rats based on haematological, serum biochemical, and histological evaluation. Thirty male Wistar rats were randomly assigned into six groups of five rats each. NaNO_2_ were given at a subacute dose of 50 mg/kg bw intraperitoneally for 10 days. Chlorophyll from* S. androgynus* and Cu-chlorophyllin from K-Liquid™ were given in the following 14 days at the two doses: 0,016 mg/mL and 0.008 mg/mL. NaNO_2_ exposure resulted in significant reductions (*p* < 0.05) in values of packed cell volume (PCV), haemoglobin (Hb) concentration and red blood cell (RBC) count, transferrin, and ferritin and elevation in malondialdehyde (MDA) level and schistocytes percentage with insignificant reductions in serum albumin and transferrin levels. Histology of kidney and liver were changed insignificantly (*p* > 0.05) to normal values. Chlorophyll from* S. androgynus* and Cu-chlorophyllin possess antioxidant potentials to protect against toxicities induced by sodium nitrate.

## 1. Introduction

Sodium nitrate (NaNO_2_) is one of important environmental toxicants and poses important health risks. Most countries use NaNO_2_ as food additive as a source of color and flavor preservation in meat products and this chemical can also be found in water resources [1]. Consumption of NaNO_2_ at low levels for long time caused histopathologic changes, nitrosative tissue damage, and lipid peroxidation in liver and kidney, as well as inducing chromosomal aberrations [2], decreased immune system [3], increased cancer colon risk [1], hypoxia, enlargement of the carotid body, and a vasodilation due to the activity with haemoglobin in the blood to form methaemoglobin, which has a much higher (up to 20 times) affinity for oxygen [4, 5]. Acute intraperitoneal treatment of Wistar rats with NaNO_2_ (at the dose of 50 mg/kg bw) influences the blood rheological properties and erythrocyte hematometric indices. As very little data are available on the effort to cure the intoxication of NaNO_2_, the aim of this work was to evaluate the curative effect of chlorophyll from* Sauropus androgynus* (L) Merr as antioxidant to cure its toxic effect in inducing oxidative stress.

Dietary chlorophyll can be found in fresh fruits and vegetables as chlorophyll a and chlorophyll b, thermally processed fruits and vegetables as metal-free pheophytins and pyropheophytins, and thermally processed green vegetables as Zn-pheophytins and Zn-pyropheophytins [6]. Chlorophyll in form of underutilized greens in fresh vegetables, supplements, liquid solutions, extracts, or tablets can be used effectively as healthy and beneficial nutrient supplement [7]. Antioxidant activity is one of the beneficial effects of chlorophylls to prevent oxidative DNA damage and lipid peroxidation both by reducing reactive oxygen species (ROS) and chelating metal ions [8–11]. Chlorophylls can act as a hydrogen donor to break the chain reaction, due to the porphyrin in its chemical structure [12].

*Sauropus androgynus* (L) Merr was identified as potentially rich sources of chlorophyll [13, 14]. The antioxidant activities of the dark green leaves of* S. androgynous* were reported to have biologically nutritive value. Among others, they have antidiabetic activity in diabetic mice induced by alloxan [15], reduce fever, increase breastmilk production, and prevent hoarse voice [16]; have antidyslipidemic activity and prevent the cardiovascular disorder in Wistar male rats induced with fat-rich diet [17]; affect the growth performances, resist diseases, and enhance nonspecific immune responses in grouper diets [18]. The previous study reported that antioxidant activity of chlorophyll from* S. androgynous* leaves is able to decrease schistocytes percentage and malondialdehyde (MDA) level and also increase the level of haemoglobin (Hb) and ferritin in female mice induced by NaNO_2_ [19]. This work may provide new information for toxicological testing to validate the safety and traditional uses of these plants.

Cu-chlorophyllin reported has a higher antioxidant activity than that of natural chlorophylls due to the presence of the chelated metal in the porphyrin ring. The antioxidant activities of the Cu-chelated compounds were found to be much higher than those of natural chlorophylls and of Mg-free derivatives [11]. This study also compared the curative effect of natural chlorophyll in* S. androgynus* leaves compared to Cu-chlorophyllin antioxidant activity in female rats induced subacute sodium nitrite (NaNO_2_). Haematological blood assays and the level of MDA, ferritin, and transferrin in blood serum were analysed as curative effect indicator of chlorophyll, while the histopathologic view of liver and kidney tissues was used to evaluate its toxicity.

## 2. Material and Methods

### 2.1. Chemicals

All chemicals were of analytical grade. Fresh leaves of* S. androgynus* (L) Merr were collected from the inhabitant park in Penggaron Lor Village, Genuk, Semarang, Central Java, Indonesia. Cu-chlorophyllin from K-Liquid was obtained from a drug store in Semarang, Central Java, Indonesia.

### 2.2. Extract Preparation of Chlorophyll from* S. androgynus* Leaves

A given amount (200 g) of fresh leaves was cleaned and was thereafter blended with acetone : methanol (7 : 3, v/v) using an electric blender until the pigment was removed. Ascorbic acid was added to the solution to avoid degradation of the pigment. The extract was filtered to remove insoluble material. The filtrate obtained was then partitioned with diethyl ether three times sequentially. The organic phase material that resulted was added steadily with Na_2_SO_4_ anhydrate to ensure no water in the extract. The solvent removal and drying process of chlorophyll used rotary evaporator and N_2_ gas. The yield of the extraction process was harvested and kept at 4°C for use. A suspension of 0.016 mg of chlorophyll from* S. androgynus* (L) Merr leaf extract (CSA) was dissolved in 1 mL distilled water, used as a dose 1 of CSA, while for making 1/2 dose CSA 0.008 mg/mL was diluted from 1 dose CSA.

### 2.3. Experimental Animals

Thirty female Wistar albino rats (150–200 g) were obtained from the Faculty of Pharmacy, Universitas Gadjah Mada, Yogyakarta, Indonesia. Rats were maintained in the animal house of Faculty of Medicine, Universitas Islam Sultan Agung in standard hard bottom polypropylene cages at 23°C ± 2°C, 12 : 12 h light/dark cycle and free access to laboratory chow and tap water throughout the study. Ethical clearance was obtained from the Ethics Committee of the Faculty of Medicine, Universitas Islam Sultan Agung, Indonesia, with document number 150/V/2015/Komisi Bioetika.

The animals were randomly divided into six groups, comprising five rats per group. All groups were acclimatized for 7 days prior to treatment. NaNO_2_ was administered 50 mg/kg body weight (dissolved in 1 mL distilled water), which refers to rate of LD_50_ on rats [20]. NaNO_2_ as much as 1 mL/day was given intraperitoneally to the rats for 10 days for treated animals (groups II, III, IV, V, and VI). The control rats (group I) were treated with the same volume of distilled water. During the following 14 days, the rats from groups III and IV were given orally dose 1/2 (0.008 mg/mL) and dose 1 (0.016 mg/mL) of CSA, respectively, whereas the rats from groups V and VI were given Cu-chlorophyllin from K-Liquid with dose 1/2 (0.008 mg/mL) and dose 1 (0.016 mg/mL), respectively. Cu-chlorophyllin was dissolved in distilled water based on the instruction in the packing. The solution of chlorophyll and Cu-chlorophyllin, those given to rat, were 3 mL/head/day according to the conversion of adult man doses.

### 2.4. Blood Sampling

Blood samples were collected from the animals via the periorbital sinus 24 h after the last treatment. About 3 mL of the blood was allowed to clot at room temperature. The clotted blood samples were centrifuged at 3000 rpm for 10 min to obtain the serum, which was used for biochemical analyses. Another 2 mL of the blood samples was collected into heparinized tubes that were used for haematological assays.

### 2.5. Haematological Blood Assays

The packed cell volume (PCV) was estimated by the microhematocrit method and the Hb concentration by the cyanmethemoglobin. Red blood cell (RBC) counts were determined using the new improved Neubauer hemocytometer. Schistocytes are detected in the peripheral blood smear stained using wedge procedures and observed by microscopy in 100x every 1000 erythrocytes.

### 2.6. Biochemical Evaluation

Commercially available kits were used according to the respective manufacturer's protocol for the measurement of MDA, ferritin, and transferrin. MDA level was measured using Thio Barbituric Acid Reactive Substance (TBARS) test with 532 nm wavelength spectrophotometer, whilst ferritin level was measured using Enzyme Linked Immunosorbent Assay (ELISA) method. The measurement of transferrin level was using automatic analyser.

### 2.7. Histological Study

All the animals were then sacrificed by anesthetizing with diethyl ether 24 h after the last treatment. The liver and kidney samples were collected in 10% formalin for histopathological analysis. The organ tissues were processed and embedded in paraffin wax and sections were made of about 4–6 *μ*m. After staining with haematoxylin and eosin, slides were examined under the microscope (Olympus, Japan) for histopathological changes and photographed. The histopathologic parameters for liver were scored as follows: (1) showing no changes or normal, (2) parenchymatous degeneration, (3) hydropic degeneration, and (4) necrosis, while the scoring for proximal tubule epithelium was as follows: (1) lesion less than 25% indicating no change, (2) lesion 25%–<50%, (3) lesion 50%–<75%, and (4) lesion more than 75% showing severe changes [21].

### 2.8. Statistical Analyses

Results are reported as mean values ± SEM and statistically analysed by One-Way ANOVA test with 95% significance level. If the data characteristics did not allow for the One-Way ANOVA test to be conducted, then the Kruskal-Wallis test became the alternative. A post hoc test was conducted where needed.

## 3. Results and Discussion

### 3.1. Haematological Evaluation

Values of haematological parameters are presented in Table 1. The Hb in induced NaNO_2_ rats group was significantly lower (*p* < 0.05) than that of rats in control groups. It indicated that the subacute administration of NaNO_2_ can cause anaemia which was indicated by the decrease of Hb level, PCV percentage, increase the RBC concentration, and schistocytes percentage. Changes in blood parameters and immune response are the direct toxic effects due to high dose administration of NaNO_2_ [22]. Hb level and PVC are considered to be most appropriate indicators of anaemia. PVC indicates the proportion of whole blood occupied by the RBC and depends on the Hb level in RBC. The percentages of schistocytes in NaNO_2_-induced rats group were significantly higher than control group which proved that NaNO_2_ is able to damage the resulting fragmented RBCs called as schistocytes. The schistocytes may have different forms such as triangular, helmet, or comma shaped of broken or fragmented erythrocytes [23]. NaNO_2_ affects the haematological and hemorheological parameters in mature rats [5, 24].

The group that was treated with one-dose chlorophyll from* S. androgynous* showed significant difference in Hb level, RBC concentration when compared with the NaNO_2_ group (*p* < 0.05), but no significant difference with the Cu-chlorophyllin in the same dose and control group. This indicated that the natural chlorophyll from* S. androgynous* leave is as effective as Cu-chlorophyllin to cure the oxidative stress caused by NaNO_2_ induction. The haematological parameters as PCV, Hb, and schistocytes in in the lower dose group of* S. androgynous* chlorophyll and Cu-chlorophyllin were not significantly different (*p* > 0.05) compared to those in the NaNO_2_ group, which indicated that lower dose of chlorophyll was not more effective at ameliorating the anaemia symptoms induced by NaNO_2_.

NaNO_2_ is one of methaemoglobin-forming drugs that may exacerbate oxidative toxicity under certain chronic or acute hemolytic settings. NaNO_2_ that will react with Hb may enhance heme- or iron-mediated toxicities [25]. The increasing of Hb level showed that NaNO_2_ induced erythrocyte methemoglobinemia by increasing reactive oxygen species (ROS) [26]. The fusion of the heme moiety of hemoglobin released from red blood cells into endothelium could provide catalytically active iron to the vasculature. Ferritin as a cytoprotection against free radicals in vitro will be increased in the increasing of ROS [27].

### 3.2. Serum Biochemistry

Table 2 presents serum biochemical parameters from six groups. The MDA level increased significantly in NaNO_2_ induction group, whereas transferrin and ferritin level were reduced significantly with exposure to NaNO_2_ when compared with control. Treatment with chlorophyll and Cu-chlorophyllin restored the values of these parameters.

ROS are important mediators of cellular degeneration. In the body, nitrate is reduced to nitrite [1] and nitrite is converted to nitrosonium ions which in turn reacts with amines and amides to form nitrosamines and nitrosamines, respectively [28]. N-nitrosamines have the ability to induce rapid oxidative stress [29] and then cause lipid peroxidation; therefore it disturbs the cellular homoeostasis [30].

The serum MDA level is one of the molecules used as indicator of lipid peroxidation to estimate oxidative stress [29]. Present study showed that rats who received NaNO_2_ for 10 days showed increasing of the MDA serum significantly compared to control group. This result is in accordance with the previous studies that elevated MDA level in NaNO_2_-given mice [31] and elevated levels of serum MDA in rats given NaNO_2_ [32]. Results from this study revealed that treatment with chlorophyll of* S. androgynus* restored MDA levels after NaNO_2_ treatments to normal values. The MDA level in group II which was not treated by the chlorophyll remained high (see Table 2). The decreasing of MDA levels in group III till group VI after the NaNO_2_ treatments is the results of the antioxidant activity of chlorophyll of* S. androgynus* and Cu-chlorophyllin. The same effects were observed for serum ferritin and transferrin. Decreased serum transferrin and ferritin levels could be a result of damage, particularly protein oxidation by reactive oxygen species generated by NaNO_2_ toxicity. There was a decrease (*p* < 0.05) in serum transferrin and ferritin in the NaNO_2_-induced rat followed by therapy with* S. androgynus* chlorophyll and Cu-chlorophyllin group compared with the control group.

### 3.3. Histopathological Evaluation

**Figure 1:**
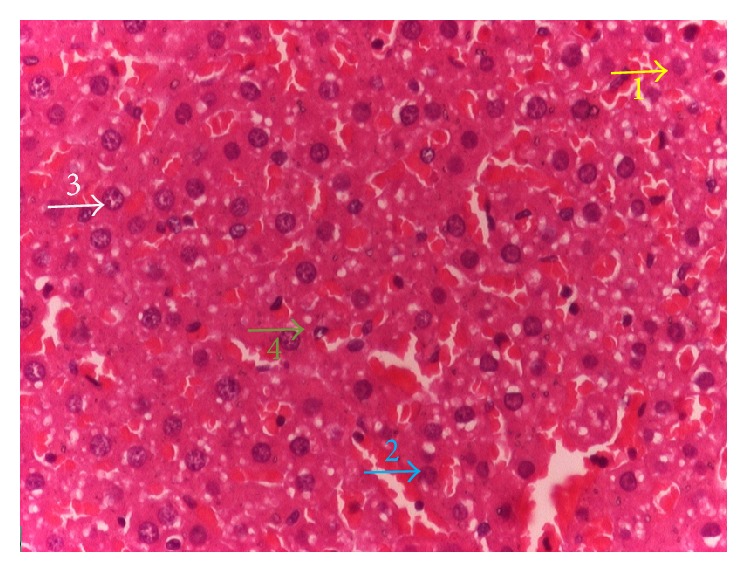
Representative microscopic tissue images of rat liver were given 50 mg/kg/day NaNO_2_ (H&E). The histopathology scores of observation are 1 (yellow arrow), 2 (blue arrow), and 3 (white arrow) showing no changes, indicating mild/moderate and severe changes, respectively. Photographs were obtained by light microscopy at 400× magnification.

**Figure 2:**
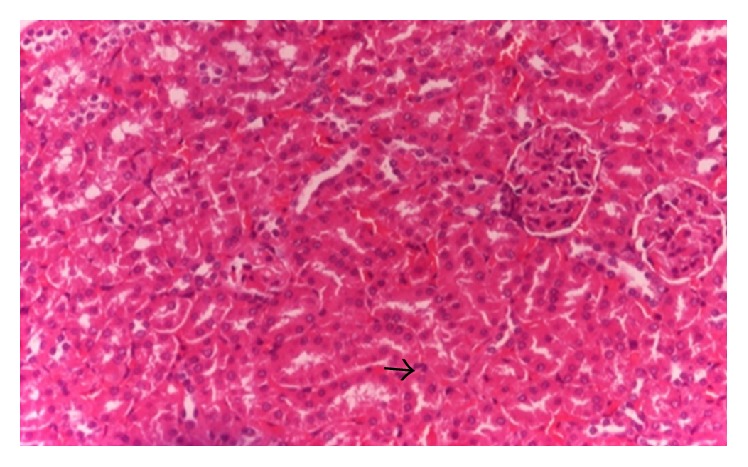
Representative microscopic tissue images of proximal tubule epithelium. Rats were given 50 mg/kg/day NaNO_2_ and 0,016 mg/mL Cu-chlorophyllin (H&E). Necrosis cell (black arrow). Photographs were obtained by light microscopy at 400× magnification.

The histopathologic parameters for liver and proximal tubule epithelium of kidney were not significantly different among groups (Table 3), although there were increasing score of hepatocyte and proximal tubule epithelium damage in NaNO_2_-induced group. Histopathology of hepatocyte of rat liver in control group showed normal or no change with score 1, while the hepatocyte in NaNO_2_ induced group showed hydropic degeneration (Figure 1). The hepatocyte and proximal tubule kidney in group treated with dose 1 Cu-chlorophyllin showed the highest score of hepatocyte histopathologic view even not significantly different from the control. There was founded necrosis cell in proximal tubule kidney of rats treated by Cu-chlorophyllin (Figure 2). The histology of kidney in the group cured with dose 1 of chlorophyll showed that the lesion cell was less than 50%.

In the present study, intake of NaNO_2_ for 10 days resulted in haematological, biochemical, and histopathological changes in rats. This result was in accordance with Roth and Kate Smith [33] that rats given NaNO_2_ via drinking water at 1–3 g/L during pregnancy and lactation showed decreasing of erythropoietic development, changing in some histopathological such as cytoplasmic vacuolization of centrilobular hepatocytes, and decreased hematopoiesis in bone marrow and spleen. Özen et al. [2] have detected hydropic cellular degeneration in liver and tubular degeneration in kidney in 20 mg/kg/day NaNO_2_-induced mice for 8 months. The histopathological changes are mediated by oxidative stress produced by the action of the metabolic pathways generated against the toxic compounds.

Lanfer-Marquez et al. (2005) reported that Cu-chlorophyllin tested by *β*-carotene bleaching method and the stable radical 2,2-diphenyl-1-picrylhydrazyl (DPPH) scavenging assay methods presented a higher antioxidant activity than that of natural chlorophylls, showing the importance of the nature of the chelated metal in the porphyrin ring. The mechanism of antioxidant activity displayed by the natural chlorophyll derivatives does not seem to be based on the ability to donate hydrogen but maybe, the protection of linoleic acid against oxidation, and/or preventing decomposition of hydroperoxides [11].

This is suggestive of hepatocytes protection from antioxidant activity from chlorophyll of* S. androgynus* against NaNO_2_-induced damages. The histopathologic parameters for liver showed NaNO_2_-induced toxicity and ameliorative potentials of chlorophyll of* S. androgynus* on NaNO_2_-induced oxidative stress.

Chlorophyll is a molecule found in the green parts of plants. Chlorophyllin copper (Cu(II)-chlorophyllin) is one of derivatives of chlorophyll that reported increased peripheral leukocyte count and improving symptoms of dizziness and fatigue in individuals with leukopenia [34]. Chlorophyll is converted to Mg-free pheophytin derivatives during digestion and then transported to peripheral tissues. Chlorophylls degraded rapidly to pheophytins in response to the high acidity of the gastric phase [35]. In the past, chlorophyll has been used to treat gastrointestinal problems, anaemia, and cancer [36]. Fahey et al. [37] reported that chlorophyll can improve the function of essential detoxification pathways. Chlorophyllin was 410-fold more potent as a phase 2 enzyme inducer than chlorophyll, since it has other detoxification properties because it is much more water-soluble than chlorophyll. Antioxidant activity of chlorophyll from plant leave also reported by Sakagami et al. [38] showed that* Sasa senanensis Rehder* leaf extract containing Fe(II)-chlorophyllin demonstrated superoxide anion and hydroxyl radical-scavenging activity five times higher than a similar product containing Cu(II)-chlorophyllin and comparable to a product containing Cu(II)-chlorophyllin, ginseng, and pine leaf extracts. Therefore, the data obtained from this study further proved the usefulness of chlorophyll of* S. androgynus* as a food supplement that may be recommended to cure humans and animals from NaNO_2_ toxicity.

## 4. Conclusion

The present study has demonstrated the curative effect of chlorophyll solution from* S*.* androgynus* leaves on haematological, serum biochemical, and histological parameters, altered by sodium nitrate exposure in female Wistar rats. This amelioration may be partly due to the antioxidant activity of chlorophyll from* S*.* androgynus* leaves that possess remarkable potential to cure the oxidative stress caused by sodium nitrate. Further assessment of molecular evaluations of biological activities of chlorophyll from* S*.* androgynus* needs to be studied.

## Figures and Tables

**Table 1:** The hematological parameters in each experimental group.

Parameters	Group I control	Group II (NaNO_2_)	Group III (NaNO_2_ + dose 1/2 CSA)	Group IV (NaNO_2_ + dose 1 CSA)	Group V (NaNO_2_ + dose 1/2 CuC)	Group VI (NaNO_2_ + dose 1 CuC)
PVC (%)	38.00 ± 2.05	36.80 ± 0.86	33.20 ± 0.80^a^	35.20 ± 1.93	38.25 ± 0.75	40.80 ± 1.85
Hb (g/dL)	13.74 ± 1.18^b^	9.26 ± 0.71^a^	10.50 ± 0.84^a^	15.32 ± 1.57^b^	13.52 ± 1.03^b^	12.84 ± 0.33^b^
RBC (×10^6^/mm^3^)	565.20 ± 14.95^b^	677.60 ± 33.62^a^	526.40 ± 49.55^b^	571.80 ± 41.49	633 ± 40.32	606.60 ± 32.83
Fragmentocyte (%)	0.02 ± 0.02^b^	0.20 ± 0.03^a^	0.10 ± 0.03	0.02 ± 0.02^b^	0.12 ± 0.06	0.16 ± 0.06^a^

Note: values are presented as mean ± SE of five animals per group. ^a^Value differs significantly from control; ^b^value differs significantly compared to NaNO_2_ alone. NaNO_2_: sodium nitrate; PCV: packed cell volume; RBC: red blood cell; Hb: haemoglobin.

**Table 2:** The serum biochemical parameters in each experimental group.

Parameters	Group I control	Group II (NaNO_2_)	Group III (NaNO_2_ + dose 1/2 CSA)	Group IV (NaNO_2_ + dose 1 CSA)	Group V (NaNO_2_ + dose 1/2 CuC)	Group VI (NaNO_2_ + dose 1 CuC)
MDA (nmol/mL)	1.26 ± 0.05^b^	4.55 ± 0.13^a^	2.88 ± 0.11^a,b^	2.13 ± 0.10^a,b^	3.35 ± 0.09^a,b^	2.53 ± 0.05^a,b^
Transferrin (%)	27.21 ± 0.18^b^	19.36 ± 0.42^a^	24.32 ± 0.06^a,b^	26.14 ± 0.03^b^	22.64 ± 0.84^a,b^	25.48 ± 0.49^a,b^
Ferritin (*μ*g/L)	113.80 ± 1.18^b^	58.20 ± 2.08^a^	75.80 ± 2.06^a.b^	93.80 ± 1.77^a,b^	65.25 ± 1.75^a,b^	86.60 ± 1.91^a,b^

Note: values are presented as mean ± SE of five animals per group. ^a^Value differs significantly from control; ^b^value differs significantly compared to NaNO_2_ alone. MDA: malondialdehyde.

**Table 3:** The degeneration score of hepatocyte and proximal tubule epithelium cell in each experimental group.

Parameters	Group I control	Group II (NaNO_2_)	Group III (NaNO_2_ + dose 1/2 CSA)	Group IV (NaNO_2_ + dose 1 CSA)	Group V (NaNO_2_ + dose 1/2 CuC)	Group VI (NaNO_2_ + dose 1 CuC)
Score of hepatocyte	1.2 ± 0.15	2.08 ± 0.23	2.04 ± 0.45	1.96 ± 0.45	1.85 ± 0.40	2.48 ± 0.39
Score of proximal tubule epithelium cell	1.08 ± 0.05	1.32 ± 0.10	1.48 ± 0.26	1.20 ± 0.09	1.25 ± 0.19	2.08 ± 0.12

Note: values are presented as mean ± SE of five animals per group.
